# Transcatheter Aortic Valve Replacement in Pure Native Aortic Valve Regurgitation: Challenging Pathology Awaiting Specialized Devices

**DOI:** 10.1055/s-0041-1725122

**Published:** 2021-10-07

**Authors:** Adam El-Gamel

**Affiliations:** 1 Cardiothoracic Unit, Waikato Hospital, Hamilton, New Zealand; 2 Department of Surgery, The University of Auckland, Auckland, New Zealand; 3 Medical Reseach Department, Waikato University, New Zealand

**Keywords:** pure native aortic incompetence, TAVR for aortic incompetence, surgical aortic valve replacement

## Abstract

Patients with aortic incompetence frequently present with anatomical and pathological challenges such as elliptical dilated annulus, dilated aortic root, dilated ascending aorta, and with no calcification in the aortic cusps or annulus. Patients are commonly in graver clinical condition as a result of a long silent clinical course before overt congestive heart failure. All of the above make transcatheter therapies for native aortic valve regurgitation more challenging with poorer outcomes, escalating the risk of insufficient anchoring, prosthesis migration, and residual paravalvular leak compared with current transcatheter aortic valve replacement (TAVR) outcomes for aortic stenosis. There is a need for specialized TAVR devices to address this complex pathology. Surgical aortic valve replacement is the current treatment option and the gold standard for patients with aortic incompetence (AR). Currently, the specific off-label indication for TAVR in pure native AR could be a feasible and reasonable option, as a compassionate treatment is limited to inoperable patients and agreed on by the heart team.

## Introduction

Transcatheter aortic valve implantation for pure native aortic regurgitation has less than optimal results compared with the current outcomes of transcatheter aortic valve replacement (TAVR) in aortic stenosis patients.

Transcatheter aortic valve implantation established itself as a noninferior treatment option compared with the gold-standard surgical aortic valve replacement (SAVR) for symptomatic patients with severe aortic stenosis. The procedure proves to be safe and efficient with excellent short-term durability. 1 2 3

**Fig. 1:**
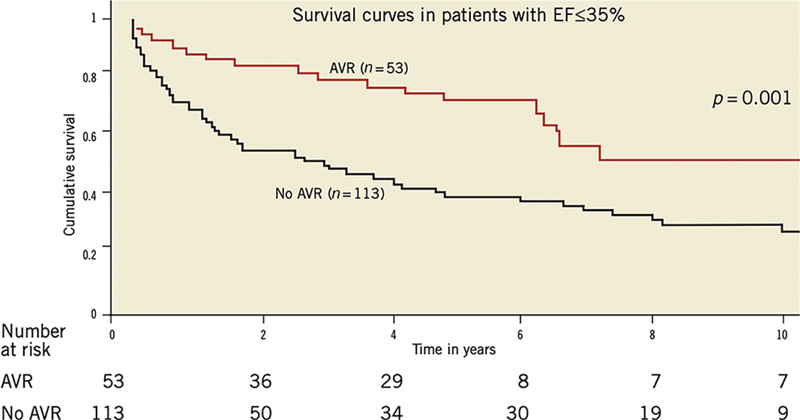
The survival of patients with severe native aortic valve regurgitation and impaired left ventricular function with and without aortic valve replacement. AVR, aortic valve replacement; EF, ejection fraction. Image Courtesy: Kamath et al. 7

SAVR is the current treatment option for patients with aortic regurgitation (AR). 2 4 The surgical treatment has excellent short- and long-term outcomes, even in patients with reduced left ventricular function. 2 5 Nonetheless, the sobering findings of the Euro Heart Survey indicated that 7.8% of patients with AR “had no intervention regardless of there was an indication for intervention,” according to the guidelines. The survey also showed that AR was present in 369 patients (13.3%) with single native left-sided valve disease, while aortic stenosis patients represented 43.1% of patients. 6 In an unselected population, the prevalence of AR in the general population was identified in the Framingham study to be 13.0% of men and 8.5% of women. All were diagnosed by echocardiography. 6 The prevalence of aortic valve incompetence rises by 2.3 times with each decade of life. 7 The choice of nonoperative treatment leads to a yearly death rate of 10 to 20% 6 which is worse for patients with poor ventricular function 7 ( Fig. 1 ).

**Fig. 2:**
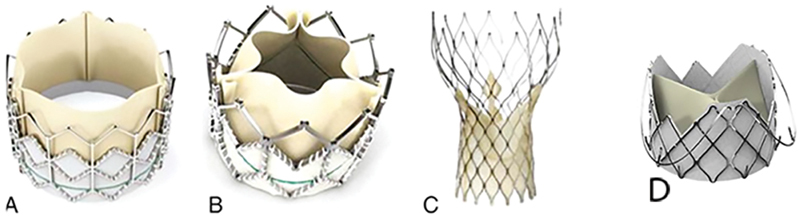
Current transcatheter aortic valve replacement valves commonly used to treat aortic incompetence patients. ( **A, B** ) Sapien, ( **C** ) CoreValve, ( **D** ) Jena-valve.

The prohibitive complications of valve migration and paravalvular leakage (PVL) preclude the widespread use of TAVR for the treatment of patients with native AR. The Jena-Valve system (Jie Cheng Medical Technology Co., Ltd., Suzhou, China) is a self-expanding transcatheter heart valve with a unique two-piece structure design ( Fig. 2 ) that comprises of three U-shaped graspers around the valve stents. The J-valve was approved for AR and aortic stenosis (AS) by China Food and Drug Administration in 2017. 8 A steep learning curve is needed for TAVR in patients with pure AR using the J-valve, which is still an off-label use in the Western world with variable and less-satisfactory outcomes compared with surgical AVR or TAVR for AS. The composite and heterogeneous pathophysiology of the aortic root complex usually requires correction of multiple components of the aortic root, than just replacing or repairing the aortic valve. It is therefore not surprising that TAVR, which only addresses the aortic valve cusps, has not achieved the desired outcomes till date. This mini review highlights the current understanding of treatment of aortic valve incompetence.

## Pathogenesis of Aortic Incompetence and Its Implication on Intervention

As clinician facing thought-provoking complex patients, we have to choose between procedures associated with suboptimal clinical outcomes versus poor prognosis without intervention. This is why the off-label uses of TAVR in the treatment of native pure AR have expanded in the last decade. 6 The pathogenesis of aortic stenosis is commonly restricted to the leaflet and the annulus of the aortic valve, 9 in contradistinction to the diverse pathology that leads to aortic regurgitation. 10 This is frequently degenerative but other pathological entities affect the function of the aortic root in patients with aortic regurgitation. Therefore, patients with AR have more intricate and inconstant anatomy. Frequently, they present with an elliptical annulus, dilated aortic root, dilated ascending aorta, or all of the above. Furthermore, the absence of calcification, the aortic cusps and the large annular size escalate the risk of insufficient anchoring, prosthesis migration, and residual PVL. All of the above makes transcatheter therapies for AR more challenging. 11 Another worry is that a fraction of AR is instigated by continued dilation of the annulus, aortic root, or ascending aorta due to aortopathy. The annual expansion of thoracic aortic aneurysms is approximately 0.3 cm 12 which increases the risk of recurrent PVL and aortic dissection. Patients with an ascending aorta aneurysm had an inadequate response to treatment with TAVR (75% of patients died within 6 months of treatment). 13 Besides, the lack of valvular calcification correlates strongly with the need for deployment of a second valve. Incidence of valve-in-valve procedures and residual AR were 30 and 88%, respectively, in the cohort reported by Testa et al, 14 pointing to the limitations of current devices for use in this specific off-label indication. 15 Yoon et al 16 published results of their review of a pure native AR, TAVR registry, and reached similar conclusions.

To add to the difficulties, patients with aortic insufficiency present frequently in grave clinical condition as a result of a long silent clinical course culminating into congestive heart failure, due to excessive volume overload, heightened left ventricular wall stress, deterioration of the ventricular function, and progressive pulmonary hypertension. All of the factors as mentioned earlier increase the risks of the procedure leaving patients with native AR more vulnerable to complications and leading to poorer outcomes compared with complex, high-risk patients with aortic stenosis. 17

At present, existing transcatheter valves are not permitted for annular measurements beyond 28 mm in diameter. Additionally, the unusual anatomy of the aortic root in patients with AR offsets the primary mechanism of action of transcatheter valves which aim to dilate the stenosed calcified valve. Malpositioning, migration, and significant PVL can only be avoided by substantial oversizing, at increased risk of annular disruption or valve dislodgment.

However, those implantations were still considered off-label, and the researchers acknowledged that there was room for improvement in device development for that challenging population.

**Fig. 3:**
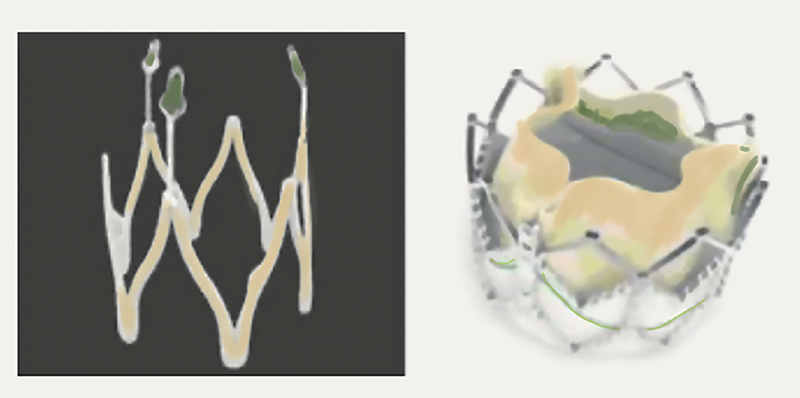
The Helio doc for the Edwards SAPIEN valve.

Attempts to produce devices specific for treating AR continued. The Helio dock (Edwards Lifesciences, Irvine, CA) is an additional device that is expected to bestow better annular fixation to the Edwards SAPIEN XT valve. 18 19 The dock is a self-expandable nitinol stent with a polyethene skirt ( Fig. 2 ) that is deployed outside and around the leaflets. This secures the balloon-expandable SAPIEN XT heart valve by including and catching the native cusps. Following the first-in-human successful implantation, a pilot trial of the procedure was performed in four patients with severe native valve AR who were deemed inoperable. 11 Although initial results were encouraging, the docking technique has not gained traction, presumably due to the complexity of the technique and lack of immediate availability of next-generation valves. At present, the manufacturer has discontinued production of the Helio dock ( Fig. 3 ).

**Fig. 4:**
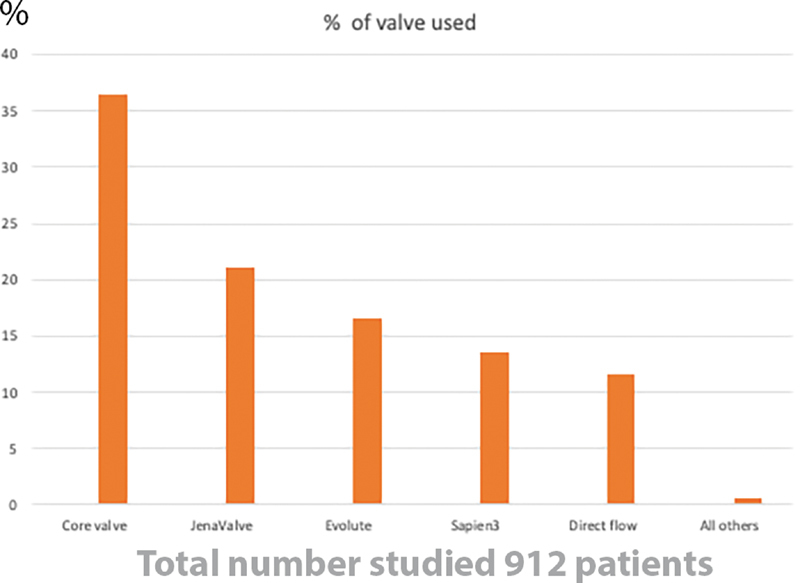
Percentage of different transcatheter aortic valve replacement valves used in treatment of native aortic valve regurgitation.

Presently, there are no transcatheter devices designated for the treatment of isolated, noncalcified native aortic valve regurgitation (NAVR). 20 The Jena valve was designed with distinctive clips and has been used off-label to treat native aortic valve AR. In 254 patients who had a procedure for NAVR, most patients were treated via a transfemoral procedure, and the most commonly used device was Corevalve ( Fig. 4 ). Contrast use was excessive. A total of 60% cases had to be done under general anesthesia. Extended hospital stay averaged 12 days, including 4 days in the intensive care unit. New pacemaker implantation was nearly 20%. 20 21

## Conclusion

Aortic regurgitation remains a challenging pathology for TAVR when “technology provides devices specifically designed to treat this complex condition,” TAVR would be “a feasible and reasonable option” for patients with pure AR. For now, there are no specifically designed devices, and TAVR for AR is compassionate treatment for inoperable patients. SAVR is still the gold-standard treatment for AR.
